# Parental satisfaction with neonatal intensive care unit services and associated factors in Ethiopia: systematic review and meta-analysis

**DOI:** 10.1186/s12912-024-01902-3

**Published:** 2024-04-08

**Authors:** Eshetu Elfios, Nefsu Awoke, Temesgen Geta, Christian Kebede, Abdulkerim Hassen

**Affiliations:** 1 Department of Nursing, School of Nursing, College of Medicine and Health Sciences, Wolaita Sodo University, Wolaita Sodo, Ethiopia; 2 Department of Nursing, School of Nursing, College of Medicine and Health Sciences, Samara University, Samara, Ethiopia

**Keywords:** Parental satisfaction, Neonatal intensive care unit, Systematic review, Ethiopia

## Abstract

**Background:**

In the context of healthcare, satisfaction is the attainment of adequate or acceptable treatment and serves as both a benchmark for quality and the ultimate objective of providing care. In neonatal care facilities, parent satisfaction is a key measure of the quality of the services offered to the newborns and aids in improving healthcare delivery. This is the first systematic review aiming to address critical knowledge gaps regarding factors influencing parental satisfaction with neonatal intensive care unit services, and determine pooled prevalence in Ethiopia.

**Methods:**

After comprehensive systematic search for full texts in the English language through an electronic web-based search strategy from databases of PubMed, CINAHL, Embase, African Journals Online, PsycINFO, and Google Scholar, included a total of 8 articles. Checklists from the Joanna Briggs Institute were used to assess the studies’ quality of methodology. STATA version 14 software for windows was used for all statistical analyses and meta-analysis was done using a random-effects method. Subgroup and sensitivity analyses were performed to clarify the source of heterogeneity.

**Results:**

Pooled national level of parents’ satisfaction with neonatal intensive unit service was 57.87% (95% CI (49.99, 65.75%)). Age of respondents and availability of chair were significantly associated with parents’ satisfaction with neonatal intensive unit service.

**Conclusions and recommendation:**

In our review we found that nearly half of parents expressed dissatisfaction with neonatal intensive unit service, which is high. Age of respondents and availability of chair in neonatal intensive unit waiting area were significantly associated with neonatal intensive unit service. Efforts to enhance parental satisfaction with neonatal intensive unit services are crucial, given that nearly half of parents reported dissatisfaction. Necessary infrastructure should be fulfilled to increase parental satisfaction with neonatal intensive unit service.

**(PROSPERO) International prospective register of systematic reviews:**

CRD42023483474

## Introduction

In neonatal care facilities, parental satisfaction is a key measure of the quality of the services offered to the newborns and aids in improving healthcare delivery [1]. Parental satisfaction is a way of responding to the expectation to meet the health needs of the people. Parental satisfaction is a belief and attitude of parents towards a specific service in an institution. It is an effective parameter in improving quality of care in neonatal intensive care unit (NICU) [2, 3].

Parent satisfaction plays a pivotal role in enhancing the quality of neonatal care. The assessment of healthcare interventions is significantly influenced by parent satisfaction, offering vital insights for evaluating staff performance, enhancing health intervention systems, facilitating future planning, and devising effective strategies for neonatal care [4].

Patient satisfaction is related to medical services; however, it is not the only factor. In the particular case of neonatal patients who are hospitalized in the NICU and cannot express their own opinion with regard to satisfaction, information is collected indirectly, from their parents. The measurement of parental satisfaction is an important indicator of the quality of services offered by an NICU, as it contributes to the evaluation of the health care provided and its improvement, resulting in the maximization of parental satisfaction with the health care system [5, 6].

Neonates are unable to communicate their health requirements, requests, experiences, opinions, or satisfaction, therefore parents play this role [7, 8]. Length of the infant’s hospital stay, Age, education level, and income are the socioeconomic factors and the parents’ sex are the most significant factors in determining how satisfied parents are [6]. Researchers found that Parents who could not hold their baby had a lower level of satisfaction. Furthermore, the parents who felt unable to protect their baby from pain and painful procedures showed the lower the degree of satisfaction [9].

Complications and morbidity often stem from inadequate quality of care, notably driven by parental dissatisfaction with NICU services and a deficiency in professional care and treatment [10]. This parental dissatisfaction can lead to inefficiencies in infant care, stemming from a shortage of essential nursing, medical, and family-centered support [11].

Furthermore, the satisfaction of parents with healthcare is linked to enhancements in their child’s health and a reduction of symptoms, encompassing adherence to the therapeutic regimen and comprehension of medical information. Consequently, the extent of client satisfaction with healthcare can serve as a valuable proxy variable, representing a crucial aspect of care quality [12].

The determinants influencing parental satisfaction included having infants outside the infection isolation room, parents with infants that breast-fed [1], parents’ educational status, parents’ occupation, duration of hospital stay, adequacy of care, and adequacy of pain management were significant factors of parental satisfaction [13].

Pooled proportion is needed to determine which sociodemographic factors are connected to parents’ satisfaction with their newborn’s care in NCU. This study can advance our understanding of parents’ satisfaction and offer advice to healthcare facilities on how to improve parents’ satisfaction in this condition.

This is the first systematic review aiming to address critical knowledge gaps regarding factors influencing parental satisfaction with neonatal intensive care unit services, and determine pooled prevalence in Ethiopia. This study will help to be used as evidence to evaluate the goal of Sustainable development goals (SDGs) planned to reduce neonatal death by improving quality of NICU services. The findings of this review will inform policy and decision makers to monitor and improve the quality of NICU services in Ethiopia. Thus, this systematic review and meta-analysis was intended to answer the following question:

What is the level of parental satisfaction with NICU services in Ethiopia, and what are the associated factors influencing parental satisfaction with the NICU service?

## Methods

### Design and search strategy

The procedure for this systematic review and meta-analysis was developed in accordance with the Preferred Reporting Items for Systematic review and Meta-analysis Protocols (PRISMA-P) statement [14]. PRISMA- 2020 statement was used to report the findings [15, 16]. This systematic review and meta-analysis was registered on PROSPERO with the registration number CRD42023483474.

We searched PubMed, CINAHL. Cochrane Library, Embase, Google Scholar, and PsycINFO database for studies reporting the level of parental satisfaction with NICU service from study conception to November 2023. We used EndNote (version X8) software to download, rearrange, review and cite the articles. Manual search was conducted for cross-references in order to find other related studies. A comprehensive search was conducted with the following search terms: “Parent satisfaction”, “satisfaction”, “determinants of parent satisfaction”, “Neonatal intensive care unit”, “NICU”, and “Ethiopia”. To combine search terms, we used Boolean operators like “AND” and “OR”.

### Eligibility criteria

We included studies reporting the level of parental satisfaction with NICU service irrespective of the type of instrument used to estimate satisfaction, the level of satisfaction assessed, and scoring system used to generate the overall score of satisfaction, Studies from both published and gray literature reported in English language, findings from national research repository, a study that has been conducted in Ethiopia, and a study that reports associated factors of parental satisfaction with NICU service were included in this review.

Studies without full text and those which lack information on Parental satisfaction with NICU service and associated factors or studies for which unable to get the necessary detail information after contacting the authors were excluded. Three authors (E.E. N.A. CK) independently evaluated the eligibility of all retrieved studies, and other reviewer’s opinion (T.G) was requested to reach a general agreement with regard to potential in- or exclusion of studies.

### Data extraction and quality assessment

Data was extracted on Microsoft Excel spread sheet. Three independent authors (EE, NA, AH) extracted the data independently. For each included article, the name of primary author, year of publication, the setting where the study was conducted or country, region, study design, study period, sample size, response rate, population, proportion of parental satisfaction and associated factors was recorded. During extraction, discrepancies between data extractors were discussed to reach agreement.

Two authors (EE, TG) independently conducted a critical appraisal of the included studies. Joanna Briggs Institute (JBI) checklists was used to assess the quality of the studies. The tool has nine parameters which have yes, no, unclear, and not applicable options (1). appropriate sampling frame (2), proper sampling technique (3), adequate sample size (4), study subject and setting description (5), sufficient data analysis (6), use of valid methods for the identified conditions (7), valid measurement for all participants (8), using appropriate statistical analysis and (9) adequate response rate [17]. The tools have yes, no, not applicable, and unknown options. One for yes responses and zero for unclear, not applicable, and no responses was scored. During the critical appraisal, whenever it was necessary other reviewers (NA, CK) were involved. Accordingly, studies that met the inclusion criteria were included and tabulated following a consensus and thorough discussion on data extraction and the completion of significant analyses by utilizing the JBI checklist. Prior to being chosen for a final review, articles undergo quality control checks. Research which scored quality index score of seven or above were categorized as low risk **(**Table 1**)**.

**Table 1:** Critical appraisal results of eligible studies in this study on parental satisfaction with NICU services and associated factors, Ethiopia, 2023

Name of author	Q1	Q2	Q3	Q4	Q5	Q6	Q7	Q8	Q9	Total
M. Seid Ali et al. [3]	Y	N	Y	Y	Y	Y	Y	Y	Y	8
A. Alemu et al. [17]	Y	Y	Y	Y	Y	Y	Y	Y	Y	9
Alle et al. [18]	Y	N	Y	Y	Y	Y	Y	Y	Y	8
Mekonen et al. [19]	Y	Y	Y	Y	Y	Y	Y	Y	Y	9
Adal et al. [20]	Y	Y	Y	Y	Y	Y	Y	Y	Y	9
Fekadu et al. [21]	Y	Y	Y	Y	Y	Y	Y	Y	Y	9
H endale [22]	Y	Y	Y	Y	Y	Y	Y	Y	Y	9
Sileshi et al. [23]	N	Y	Y	Y	Y	Y	Y	Y	Y	8

*Y* Yes, *N* No, JBI critical appraisal checklist for studies reporting prevalence data: Q1 = was the sample frame appropriate to address the target population? Q2-Were study participants sampled appropriately? Q3-Was the sample size adequate? Q4-Were the study subjects and the setting described in detail? Q5-Was the data analysis conducted with sufficient coverage of the identified sample. Q6-Were the valid methods used for the identification of the condition? Q7-Was the condition measured in a standard, reliable way for all participants? Q8-Was there appropriate statistical analysis? Q9-Was the response rate adequate, and if not, was the low response rate managed appropriately?

### Data analysis methods

The extracted data were imported to STATA 14 statistical software was used to carry out the pooled proportion of parental satisfaction with NICU service in Ethiopia. A meta-analysis of the level of parental satisfaction with NICU care was carried out using a random-effects method since it is the most common method in a meta-analysis to adjust for the observed variability [24].

To examine the possible risk of publication bias and small study effects, funnel plots and Egger’s test was used [25, 26]. Cochrane Q-Static and I^2^ were used to confirm heterogeneity between studies. Subgroup analyzes were performed to compare the pooled prevalence of parental satisfaction with NICU service and associated factors across regions. Pooled prevalence was presented in forest pilot format with 95% CI.

## Results

### Search result and study characteristics

**Fig. 1:**
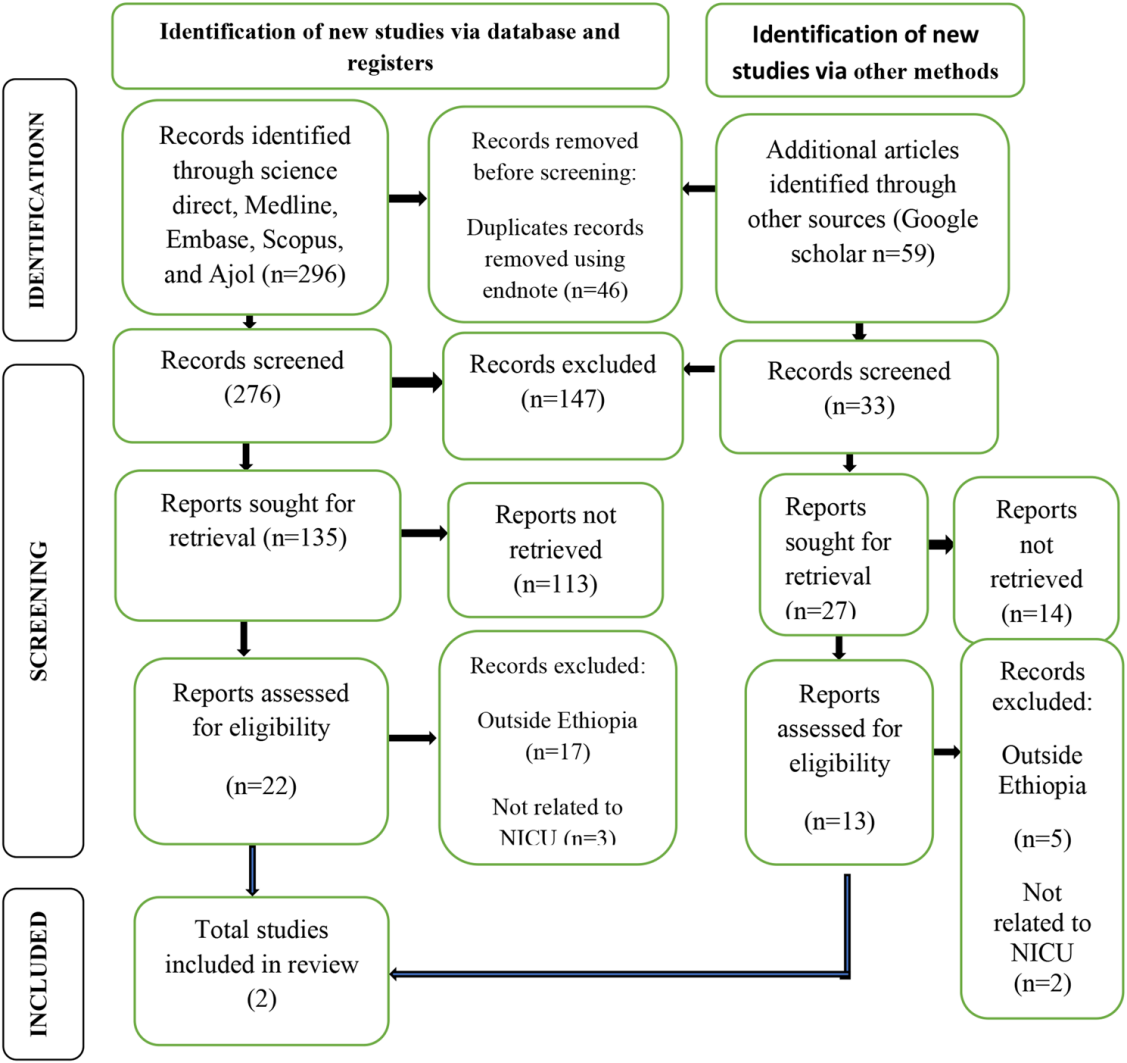
PRISMA flow diagram of the selection process of studies on parental satisfaction with NICU service in Ethiopia, 2024

The electronic online search from database searching of PubMed, Google scholar, CINAHL, African Journals Online and manual search yielded 354 records, of which 46 duplicate records were identified and removed. Title and abstract screening resulted in the exclusion of 147 irrelevant articles. Then, remaining 162 articles underwent for full-text review. Among these, 154 articles were excluded based on the predetermined eligibility criteria. Finally, a total of 8 articles were included in the meta-analysis (Fig. 1).

A total of 8 studies with 2255 participants were included in this meta-analysis. Of these four studies were conducted in Amhara region of Ethiopia [13, 19, 27, 28], Two studies were conducted in Oromia region of Ethiopia [20, 21], One study was conducted in Addis Ababa [22] and remaining one study was conducted in Southern Ethiopia [17]. All these included studies were cross-sectional in design and sample sizes ranged from 109 [21] to 401 [17] (Table 2).

**Table 2:** Characteristics of studies included in the meta-analysis of parent satisfaction with NICU service

Name of author	Year	Region	Health facility name	Study design	Sample size	Proportion of satisfied patients % (95%CI)
M. Seid Ali et al. [3]	2021	Amhara	Gondar specialized Hospital	Cross-sectional	317	50% (95% CI = (0.19–0.65)
A. Alemu et al. [17]	2022	Amhara	Public hospitals in Bahirdar	Cross-sectional	400	55% (95% CI: 50.0–59.9)
Alle et al. [18]	2022	Amhara	Debre Tabor specialized hospital	Cross-sectional	385	47.8% (95% CI= (43.1–52.5)
Mekonen et al. [19]		Amhara	Debre Birhan referral hospital	Cross-sectional	127	77% (95% CI:70–84)
Adal et al. [20]	2022	Oromia	Jimma university medical center	Cross-sectional	114	57.9% (95% CI: (49, 66%)
Fekadu et al. [21]	2022	Oromia	Primary hospitals of Jimma zone	Cross-sectional	109	72.75 (95% CI:64, 81)
H. endale [22]	2017	Addis Ababa	Selected governmental hospital in Addis Ababa	Cross-sectional	400	41.8% (95%CI: 37, 47)
Sileshi et al. [23]	2023	South Ethiopia	Referal hospitals of South Ethiopia	Cross-sectional	401	63% ((95% CI: 58%, 68%)

### Patient satisfaction with nursing care

**Fig. 2:**
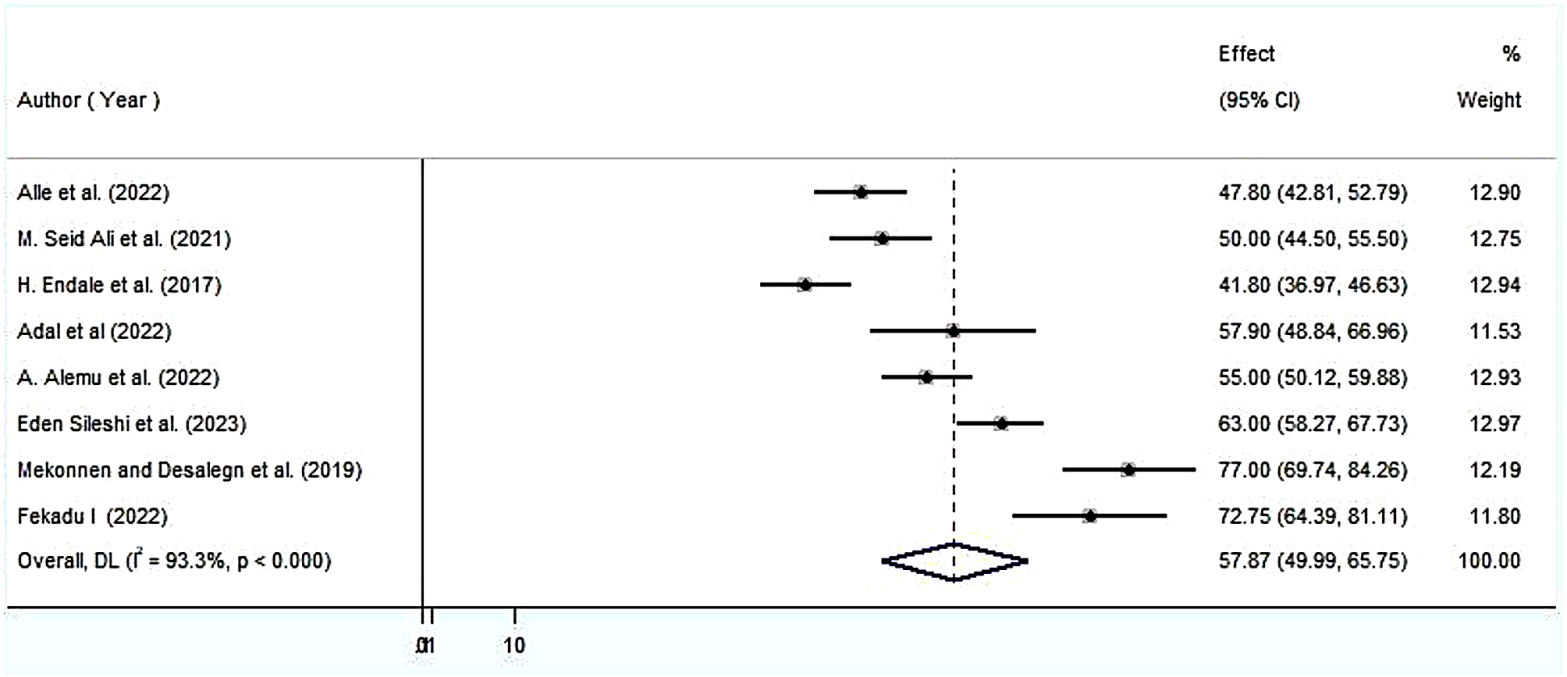
Forest plot showing the pooled level of satisfied parents with NICU service

The pooled effect size of parental satisfaction with NICU service using the fixed effect model showed significant heterogeneity across the studies. Therefore, we performed the analysis with a random effects model with 95% CI in order to adjust for the observed variability. Accordingly, the pooled national level of parents’ satisfaction with NICU service was 57.87% (95% CI (49.99, 65.75%)) with significant heterogeneity between studies (I^2^ = 93.3, *P* = 0.000) (Fig. 2).

**Fig. 3:**
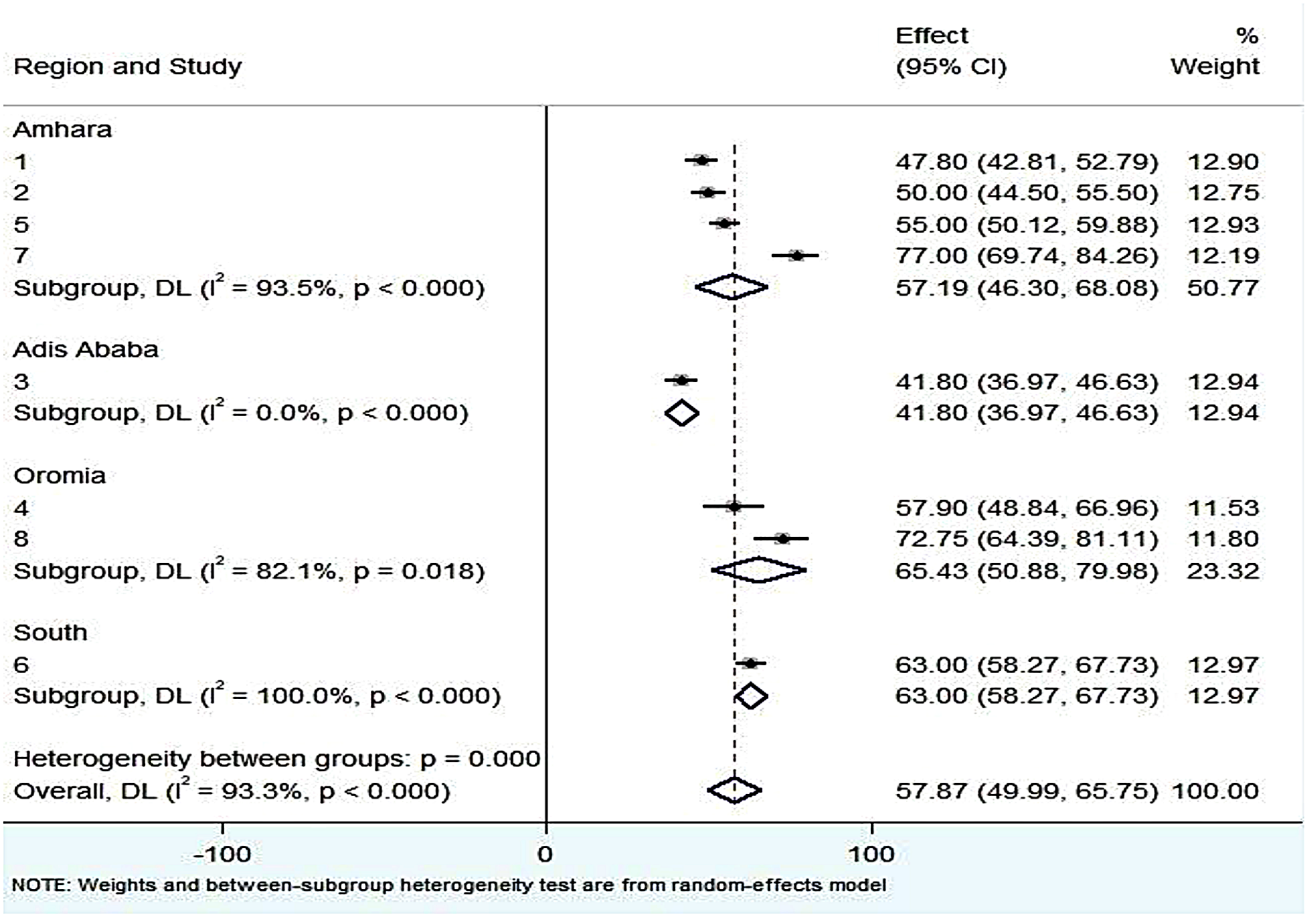
Subgroup analysis by regions on the level of parental satisfaction with NICU service

Based on the subgroup analysis by region, the highest level of parental satisfaction was observed in Oromia (65.43% (95% CI: 50.88, 79.98), I^2^ = 82.1%) while, the lowest level of parental satisfaction was observed in Addis Ababa (41.80% (95% CI: 36.97, 46.63), I^2^ = 0.0%) (Fig. 3).

### Publication bias and heterogeneity

**Fig. 4:**
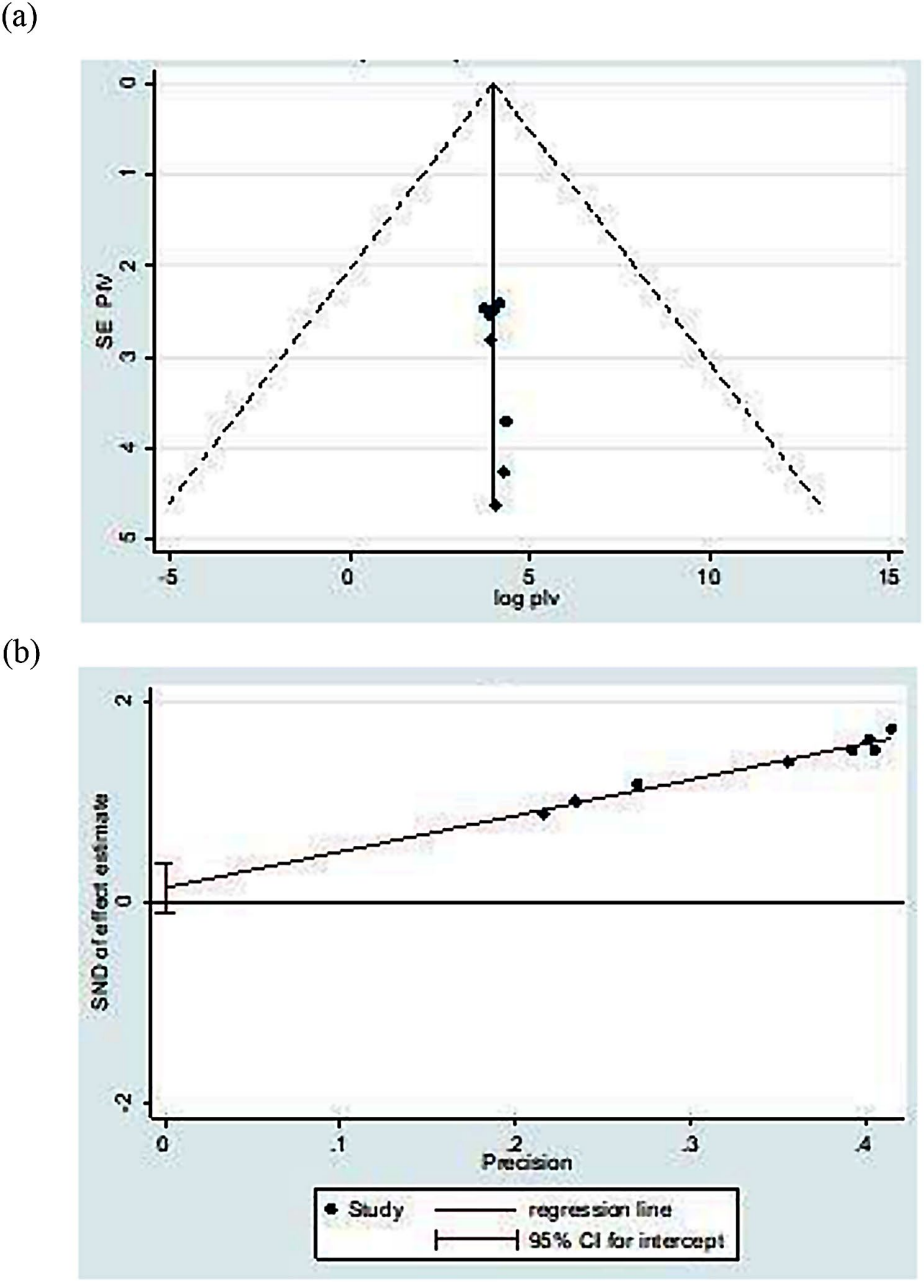
(**a**) funnel plot and (**b**) Egger’s test of the study

Presence of publication bias was examined using visual inspection of the funnel plot and Egger’s test. Visual inspection of the funnel plot suggested symmetrical distribution of included studies (Fig. 4a). The result of Egger’s test was not statistically significant for the presence of publication bias (*P* = 0.174) (Fig. 4b). Both tests confirm that there was no publication bias. The result of this meta-analysis revealed statistically significant heterogeneity among studies (I^2^ = 93.3%), we performed a subgroup analysis by region to adjust and minimize heterogeneity in addition to exploring potential sources of heterogeneity and examining whether the effect size varies across different subgroups. (Fig. 3).

Additionally, in our efforts to pinpoint potential sources of heterogeneity, we conducted a meta-regression analysis, incorporating sample size and publication year as covariates. Nevertheless, our findings revealed that neither of these variables had a significant impact on the observed heterogeneity among studies (Table 3).

**Table 3:** Meta-regression analysis of factors affecting between-study heterogeneity

Heterogeneity source	Coefficients	Std. Err.	P - value
Publication year	1.388396	1.785593	0.472
Sample size	− 0.0626259	0.0264388	0.064

### Leave-out-one sensitivity analysis

**Fig. 5:**
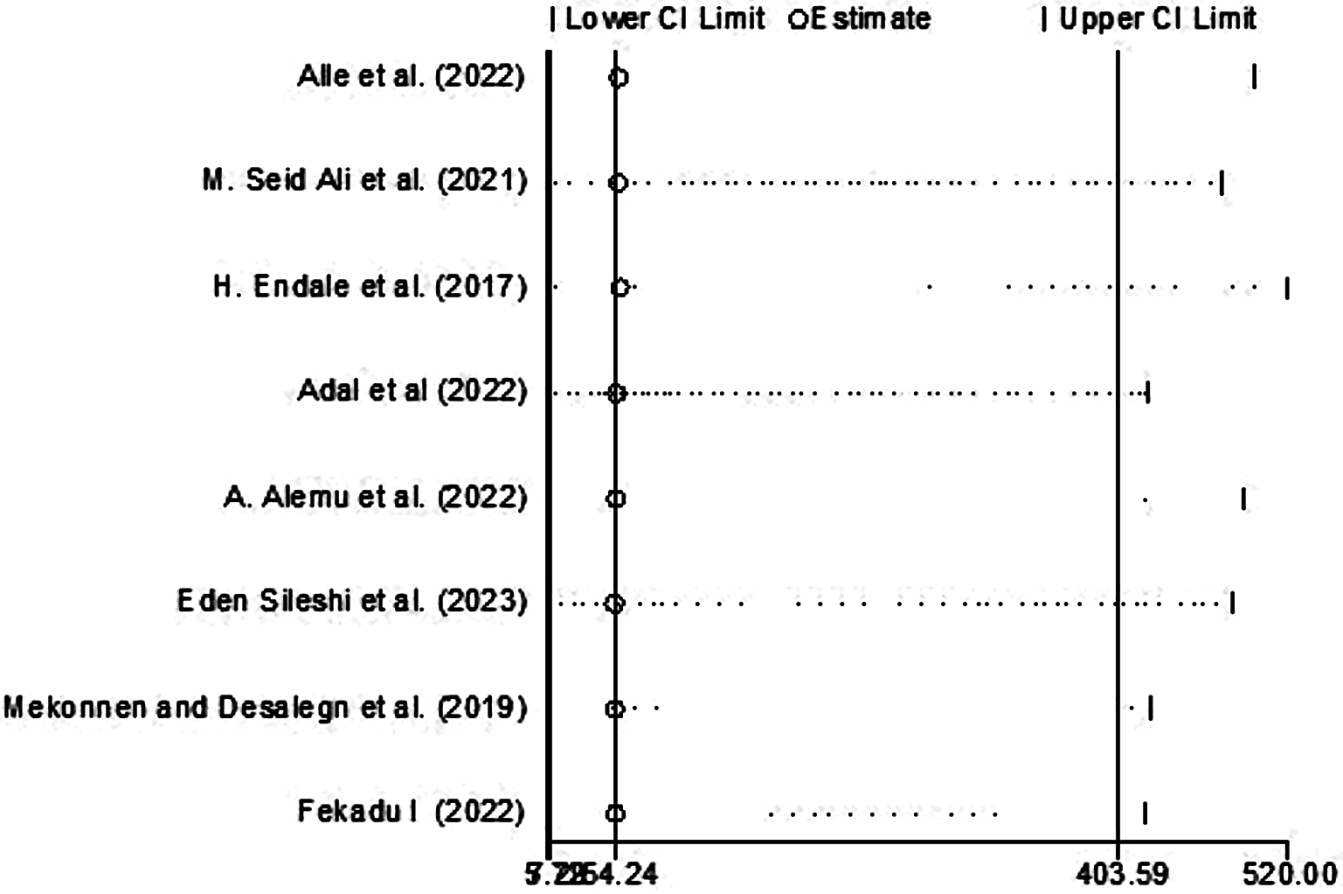
Sensitivity analysis of pooled prevalence for each study being removed at a time for systematic review and meta-analysis of turnover intention among nurses in Ethiopia

The leave-out-one sensitivity analysis conducted t assess the effect of a single studies on the overall pooled proportion of parental satisfaction with NICU service. In this systematic review, each study was excluded from the analysis one at a time. The outcomes of this analysis indicated that the exclusion of any single study did not lead to a statistically significant alteration in the overall pooled associated factors with parental satisfaction with NICU service Ethiopia. The findings are visually represented in Fig. 5, illustrating the stability of the overall pooled estimate even with the removal of specific studies from the analysis.

### Associated factors with parental satisfaction with NICU service

**Fig. 6:**
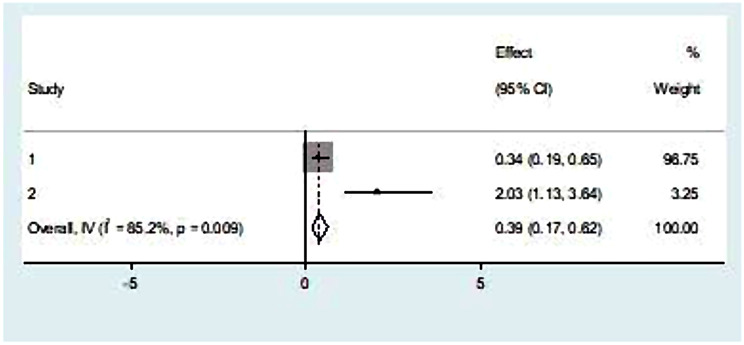
Pooled effect (OR) of the association between age and parental satisfaction on NICU service in Ethiopia, 2023

**Fig. 7:**
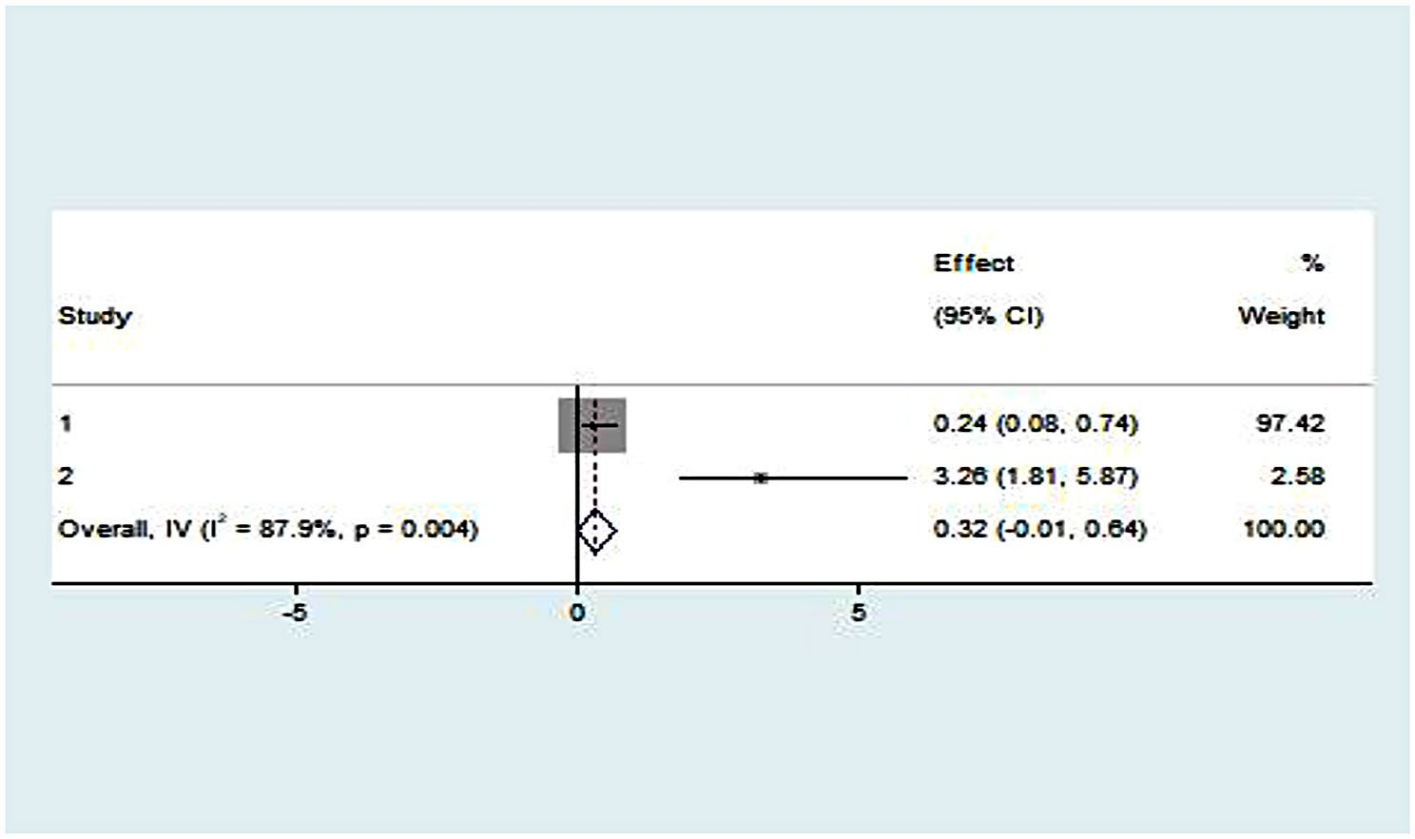
Pooled effect (OR) of the association between availability of chair and parental satisfaction on NICU service in Ethiopia, 2023

In our review we found that two variables (age of respondents and availability of chair) were significantly associated with NICU service. Parents in the age group of 25–35 had 61% lower odds of satisfaction compared to the reference group of older age categories (OR = 0.39, *p* = 0.009, I^2^ = 85.2%) (Fig. 6). In our review, the availability of a chair for parents was significantly associated with 3.13 times increase in parental satisfaction (OR = 0.32, *p* = 0.004, I^2^ = 87.9%). (Fig. 7).

## Discussion

Using data from both published and unpublished studies, this meta-analysis was carried out to determine the degree of parents’ satisfaction with NICU service nationwide and to identify the factors associated to it. This meta-analysis showed that Ethiopian parents’ satisfaction with NICU service was 57.87% (95% CI (49.99, 65.75%)) with significant heterogeneity between studies (I^2^ = 93.3, *P* = 0.000). This result was consistent with earlier research carried out in London 56% [29]. However, our meta-analysis’s assessment of parents’ satisfaction with NICU service was lower than what other comparable studies reported USA(Massachusetts and California), 21 European union countries and Norway [8, 30, 31]. The difference in the hospital infrastructure and socioeconomic status could be the cause of this discrepancy. Developed nations offer higher quality healthcare than developing nations. Healthcare organizations in industrialized nations incorporate technologies into their operational structure and patient interaction strategy to enhance the overall parent satisfaction. The parental satisfaction in this review is higher than the study conducted in Greece 48.7% [1]. This difference might be due to variations in the measurement tools used.

According to our subgroup analysis by region, Oromia had the highest degree of parental satisfaction (65.43% (95% CI: 50.88, 79.98). This might be due to Oromia region has a greater nurse to neonate ratio than other parts of the nation. However, parents in Addis Ababa, the capital city, may be more knowledgeable and have higher expectations. If these expectations are not met, this could lead to a decrease in satisfaction [32].

In our review we found that age of parents and availability of chair were significantly associated with parental satisfaction with NICU service. Other socio-demographic as well as parent and newborn related factors were found non-significant in final meta-analysis.

On this systematic review and meta-analysis we found that parents in the age group of 25–35 had 61% lower odds of satisfaction compared to the reference group of older age categories This finding is in line with the study conducted in Greece [4]. Because they are less experienced, younger parents could have lower needs and expectations from the healthcare providers in NICU. While older parents having higher expectations from healthcare providers and when this expectation met, they satisfy with the care given to their newborns. In contrary to this finding, study conducted in Norway revealed that age of parents was not significantly associated with overall parental satisfaction [31].

Our review revealed a significant association between the availability of a chair for parents and parental satisfaction in neonatal care units. The odds ratio of 0.32 (95% CI: 0.15–0.68, *p* = 0.004) indicates a substantial impact, suggesting that parents who had access to a chair were approximately 3.13 times more likely to report higher satisfaction levels compared to those without such amenities. This finding underscores the significance of environmental factors in healthcare settings, particularly those catering to neonatal care. Comfortable facilities, such as the availability of seating for parents, play a crucial role in shaping their overall satisfaction with the care provided. This aligns with previous studies emphasizing the importance of a supportive and accommodating environment in healthcare settings [33].

## Conclusion and recommendation

Based on the outcomes of our systematic review and meta-analysis, which revealed a low proportion of parental satisfaction with NICU services, we recommend healthcare facilities to address a range of factors influencing parental satisfaction comprehensively. Beyond the provision of seating arrangements, it is crucial to explore thoroughly the impact of age and regional variations on parental satisfaction. Future research endeavors should focus on clarifying these complexities further, enabling the development of tailored interventions. By addressing existing knowledge gaps, we can enhance our understanding of the intricate dynamics influencing parental satisfaction in neonatal care units, ultimately contributing to more effective and targeted healthcare strategies.

The strength of this study lies in its extensive systematic review and meta-analysis, reaching across varied regions. It thoroughly explores the complex factors that impact parental satisfaction in neonatal care units, offering valuable insights for better interventions and policy enhancements. Our review had few limitations, even though it has given useful information and current evidence on the degree of parent satisfaction with NICU service in Ethiopia. Careful consideration must be given to the interpretation of the results as our aggregate estimations revealed considerable variation among the research.

## Data Availability

The datasets generated and/or analyzed during the current study are available from the corresponding author upon reasonable request.

## Abbreviations

AJOL: African Journals Online
CINAHL: Cumulative Index to Nursing and Allied Health Literature
JBI: Joanna Briggs Institute
MeSH: Medical Subject Headings
NICU: Neonatal Intensive Care Unit
PRISMA-P: Preferred Reporting Items for Systematic Review and Meta-Analysis Protocols
